# Determinants of HIV infection among adolescent girls and young women aged 15–24 years in South Africa: a 2012 population-based national household survey

**DOI:** 10.1186/s12889-018-5051-3

**Published:** 2018-01-26

**Authors:** Musawenkosi Mabaso, Zinhle Sokhela, Neo Mohlabane, Buyisile Chibi, Khangelani Zuma, Leickness Simbayi

**Affiliations:** 1 Epidemiology and Strategic Information Unit, HIV/AIDS, STIs and TB programme, Human Sciences Research Council, PO Box 37429, Overport, Durban, 4067 South Africa; 2 HIV/AIDS, STIs and TB programme, Human Sciences Research Council, Port Elizabeth, South Africa; 3 HIV/AIDS, STIs and TB programme, Human Sciences Research Council, Pretoria, South Africa; 4 Research Methodology and Data Center, Human Sciences Research Council, Pretoria, South Africa; 5 Office of the Deputy CEO for Research, Human Sciences Research Council, Cape Town, South Africa; 6 Department of Psychiatry & Mental Health, University of Cape Town, Cape Town, South Africa

**Keywords:** Determinants, HIV infection, Adolescent girls, Young women, South Africa

## Abstract

**Background:**

South Africa is making tremendous progress in the fight against HIV, however, adolescent girls and young women aged 15–24 years (AGYW) remain at higher risk of new HIV infections. This paper investigates socio-demographic and behavioural determinants of HIV infection among AGYW in South Africa.

**Methods:**

A secondary data analysis was undertaken based on the 2012 population-based nationally representative multi-stage stratified cluster random household sample. Multivariate stepwise backward and forward regression modelling was used to determine factors independently associated with HIV prevalence.

**Results:**

Out of 3092 interviewed and tested AGYW 11.4% were HIV positive. Overall HIV prevalence was significantly higher among young women (17.4%) compared to adolescent girls (5.6%). In the AGYW model increased risk of HIV infection was associated with being young women aged 20–24 years (OR = 2.30, *p* = 0.006), and condom use at last sex (OR = 1.91, *p* = 0.010), and decreased likelihood was associated with other race groups (OR = 0.06, *p* < 0.001), sexual partner within 5 years of age (OR = 0.53, *p* = 0.012), tertiary level education (OR = 0.11, *p* = 0.002), low risk alcohol use (OR = 0.19, *p* = 0.022) and having one sexual partner (OR = 0.43, *p* = 0.028). In the adolescent girls model decreased risk of HIV infection was associated with other race groups (OR = 0.01, *p* < 0.001), being married (OR = 0.07), *p* = 0.016], and living in less poor household (OR = 0.08, *p* = 0.002). In the young women’s models increased risk of HIV infection was associated with condom use at last sex (OR = 2.09, *p* = 0.013), and decreased likelihood was associated with other race groups (OR = 0.17, *p* < 0.001), one sexual partner (OR = 0.6, *p* = 0.014), low risk alcohol use (OR = 0.17, *p* < 0.001), having a sexual partner within 5 years of age (OR = 0.29, *p* = 0.022), and having tertiary education (OR = 0.29, *p* = 0.022).

**Conclusion:**

These findings support the need to design combination prevention interventions which simultaneously address socio-economic drivers of the HIV epidemic, promote education, equity and access to schooling, and target age-disparate partnerships, inconsistent condom use and risky alcohol consumption.

## Background

Globally, 80% of adolescent girls and young women (AGYW) aged 15–24 years living with HIV are in sub-Saharan Africa (SSA). In addition, the number of new HIV infections among AGYW in SSA remains exceptionally with about 450,000 [380000–530,000] new infections estimated in 2015 [1]. Eastern and Southern Africa countries carry the heaviest burden with an estimated 236,000 annual number of new infections, and South Africa leading with about 102,000 new infections in the same year [1, 2]. This is the case despite significant investment and gains made to reduce HIV risk over the past decades in the country [3]. Some of the AGYW acquire HIV perinatally while many are infected through heterosexual sexual intercourse.

Factors that contribute to the high prevalence and incidence of HIV among AGYW are varied and may differ by sub-region, country, and context. Vulnerability of AGYW to HIV is affected by societal norms supportive of male superiority and sexual entitlement. Evidence shows that this leads to gender inequality and unequal power dynamics causing females to be unable to negotiate safe sex leading them to engage in risky sexual behaviours [4, 5]. High risk behaviours associated with HIV among AGYW include early sexual debut, multiple sexual partnerships, limited condom use, intimate partner violence, intergenerational and transactional sex [6–10]. Furthermore, socio-demographic factors such as age, marital status, level of education, employment, and place of residence have also been associated with risk of HIV among young people [11, 12].

In addition, in many sub-Saharan countries including South Africa, poverty is a major concern in most families, and due to family’s economic deprivation AGYW are often expected fend for themselves and to contribute financially or materially to support their families from a young age, which discourages adolescent girls to enrol and or/stay in school [13, 14]. Consequently, limited economic options that are available to AGYW due to low/no education strongly influence their decisions to engage in unsafe sexual behaviour [13, 14].

Studies have shown that social, demographic and behavioural factors interact in different ways to increase risk of HIV among AGYW under different settings in SSA [14]. Improved understanding of the determinants of HIV among AGYW given the changing epidemic in South Africa will provide a platform for new and highly targeted impactful intervention strategies to prevent and reduce HIV infection among AGYW in the country. This paper investigates socio-demographic and behavioural determinants of HIV infection among AGYW aged 15–24 years in South Africa.

## Methods

### Study design and sample

This analysis is based on the 2012 South African National HIV Prevalence, Incidence, and Behaviour Survey, a nationally representative population-based household survey, described in detail elsewhere [3]. Study participants were selected using multi-stage stratified cluster sampling. A systematic probability sample of 15 households was drawn from each of 1000 enumeration areas (EAs) selected randomly from strata defined by locality type, race group, and province as defined by the census in South Africa. A detailed questionnaire soliciting information related to: demographics, HIV-related attitudes, practice, behaviours, and knowledge was administered.

Detailed age appropriate questionnaires soliciting information related to: demographics, HIV-related attitudes, practice, behaviours and knowledge were administered. Apart from collecting behavioural survey data, dried blood spots (DBS) specimens were also collected from participants who consented for HIV testing. Samples were tested for HIV using an enzyme immunoassay (EIA) (Vironostika HIV Uni-Form II plus O, Biomeriux, Boxtel, The Netherlands), and samples which tested positive were retested using a second EIA (Advia Centaur XP, Siemens Medical Solutions Diagnostics, Tarrytown, New York, USA). Any samples with discordant results on the first two EIAs were tested with a third EIA (Roche Elecys 2010 HIV Combi, Roche Diagnostics, Mannheim, Germany).

### Measures

This analysis focused socio-demographic and HIV related risk behaviours only on AGYW aged 15–24 years. The primary outcome measure is HIV status (i.e., 0 = HIV negative, 1 = HIV positive). Socio-demographic measures included age (15 to 19 years and 20 to 24 years), race (African Black or other), marital status (not married or married), education level (no education/primary, secondary, tertiary), employment status (unemployed or employed), and locality type (urban formal, urban informal, rural informal, rural formal), and asset based socio-economic status (SES) constructed using multiple correspondence analyses (MCA) based on questions on availability/ownership of broad range of household assets ownership and access to utilities. MCA calculated a composite indicator score computed by adding up all weighted responses [15]. The predicted score for each household was used to compute five quintiles (1st lowest, 2nd lower, 3rd middle, 4th higher and 5th highest) representing a continuum of household SES from the most poor to the least poor.

Risk behaviours included the age at sexual debut (less than 15 years or more than 15 years), age-disparate sexual partnership (The categories should be: partner 5 years and older, partner 5 years and younger, partner within 5 years older or younger), multiple sexual partners in the last 12 months (one partner or two or more sexual partners), condom use during sexual contact (consistent or inconsistent), self-perceived risk of HIV infection (no or yes), and alcohol use risk score (0 = abstainers; 1–7 = low-risk drinkers; 8–19 = high-risk drinkers; 20+ = hazardous drinking) based on a questionnaire for Alcohol Use Disorder Identification Test (AUDIT) [16].

### Data analysis

All statistical analysis was done in STATA 12 software using *svy* commands to take into account the complex multi-level survey design (Stata Corporation, College Station, Texas, USA). Data were weighted to account for the differential selection probabilities at the enumeration areas, households, and individual levels. Final weights were benchmarked to the official Statistics South Africa national midyear population estimates by age, race, sex and province to ensure that the data was nationally representative.

Descriptive statistics was used to summarize socio-demographic and sexual risk behaviour characteristics of the study sample, and differences between categorical variables was assessed using Pearson chi-square test of independence. Multivariate logistic regression analysis using a combination of backward and forward selection procedures was employed to determine factors independently associated with HIV prevalence. Three models were fitted for (1) AGYW 15–24 years (2) adolescent girls 15–19 years and (3) young women 20–24 years. Probabilities for removal and entry of variables into the models were set at *p*-values of 0.20 and 0.10. Coefficient plots were used to display the results of the final models. Odds ratios (ORs) with 95% confidence intervals (CI) are reported, and *p*-values less than 0.05 were considered statistical significant.

## Results

### Socio-demographic characteristics and HIV prevalence

Table 1 describes the study sample and shows HIV prevalence by socio-demographic characteristics. The majority of the young women were aged between 20 and 24 years (51.1%), were Black African (83.29%), not married (95.5%), had secondary education (87.3%) and resided mainly in urban formal setting (49.8%), and were from poor households in the 1st (25.1%) and 2nd (26.6%) quintiles.. The overall HIV prevalence in this sample of young women was 11.4% (95% CI, 9.8–13.2) (*n* = 3092). HIV prevalence was significantly higher among young women aged between 20 and 24 years (17.4%, *p* < 0.001) and Black Africans (13.5%, *p* < 0.001), those residing in urban informal areas (19.1%, *p* = 0.007), and those from poor households in the 1st (13.2%) and 2nd (16.7%) quintiles (*p* < 0.001).

**Table 1:** HIV prevalence by socio-demographic characteristics of the study sample (*n* = 3092)

Variables	Totals^*^	%	HIV Prevalence	95% CI	*p*-value^**^
Age					
15 to 19 years	1606	51.1	5.6	4.2–7.5	< 0.001
20 to 24 years	1486	48.9	17.4	14.6–20.6	
Race					
Black African	2130	83.2	13.5	11.6–15.7	< 0.001
Other	955	16.8	0.9	0.4–1.7	
Marital status					
Not Married	2874	95.5	11.7	10.0–13.7	0.303
Married	140	4.5	7.2	2.8–17.2	
Education level					
Primary school	252	7.8	16.7	10.3–25.9	0.121
Secondary school	2406	87.3	11.4	9.6–13.5	
Tertiary	126	4.8	6.4	2.6–15.2	
Employment					
Unemployed	2604	88.0	11.3	9.5–13.3	0.651
Employed	363	12.0	12.8	7.6–20.8	
Locality					
Urban formal	1578	49.8	9.2	7.2–11.8	0.007
Urban informal	367	7.3	19.1	13.3–26.8	
Rural informal	891	39.5	12.7	10.0–16.1	
Rural formal	256	3.5	10.3	7.1–14.8	
Assert based SES^***^					
Quintile 1	705	25.1	13.2	10.0–17.1	< 0.001
Quintile 2	681	26.6	16.7	12.9–21.4	
Quintile 3	642	21.4	11.5	8.1–16.2	
Quintile 4	684	19.2	6.5	3.9–10.6	
Quintile 5	329	7.8	3.0	0.9–9.4	

^*^Totals do not add to overall total due to missing data, ^**^p < 0.05 considered significant, ^***^Quintiles 1–5 represent a continuum of household socio-economic status (SES) from the most poor to the least poor

### Behavioural characteristics and HIV prevalence

Table 2 describes the study sample and shows HIV prevalence by behavioural characteristics. The overwhelming majority of AGYW reported that they started having sex at age 15 years and above (96.5%) and had two or more sexual partners (91.4%) while large majorities indicated that they had same age partners (63.9%), engaged in inconsistent condom use (63.4%), thought they were at risk of HIV (78.6%), and were abstainers (76.7%). HIV prevalence was significantly higher among AGYW who perceived themselves as not at risk of HIV (20.8%, *p* < 0.001), and abstainers among alcohol users (12.6%. *p* = 0.034).

**Table 2:** HIV prevalence by behavioural characteristics of the study sample (*n* = 3092)

Variables	Total^*^	%	HIV prevalence	95% CI	*p*-value^**^
Age at sexual debut (years)					
< 15	105	3.5	10.0	3.5–25.4	0.802
> =15	2976	96.5	11.4	9.8–13.3	
No of sex partners in last 12 months					
One partner	1395	8.6	15.5	12.8–18.6	0.100
2 or more partners	127	91.4	22	13.8–33.2	
Age-disparate sexual partnership					
Partner 5 years and older	565	36.1	18.0	13.6–23.4	0.060
Partner 5 years and younger	2	0.1			
Partner within 5 years older or younger	951	63.9	14.8	11.5–18.9	
Condom use during sexual contact					
Consistent	564	36.4	18.6	14.1–24.0	0.534
Inconsistent	944	63.4	14.9	11.8–18.6	
Self-perceived risk of HIV Infection					
No	615	21.4	18.9	14.9–23.8	< 0.001
Yes	2438	78.6	9.3	7.6–11.3	
Alcohol use risk score (AUDIT)^***^					
Abstainers	2095	76.7	12.6	10.6–14.9	0.034
Low risk (1–7)	518	18.4	8.8	5.4–14.2	
High risk drinkers (8–19)	132	3.6	3.0	1.2–7.1	
Hazardous drinkers (20+)	13	0.8	9.2	2.2–31.3	

^*^Totals do not add to overall total due to missing data, ^**^p < 0.05 considered significant ^***^Alcohol risk score based on a questionnaire for Alcohol Use Disorder Identification Test (AUDIT)

### Multivariate models

**Fig. 1:**
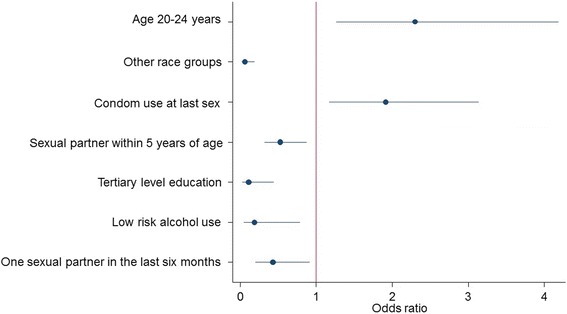
Multivariate logistic regression model (significant at *p* < 0.05) of the determinants of HIV among adolescent girls and young women (15–24 years)

Figure 1 presents a combination of factors independently associated with HIV prevalence among AGYW 15–24 years. In this model increased likelihood of HIV infection was significantly associated with young women aged 20–24 years than adolescent girls aged 15–19 years [OR = 2.30 (95% CI: 1.26–4.18), *p* = 0.006], and reported condom use at last sex [OR = 1.91 (95% CI:1.17–3.14), *p* = 0.010)]. Decreased likelihood of HIV infection was significantly associated with other race groups than Black Africans [OR = 0.06 (95% CI: 0.02–0.19), *p* < 0.001, having a sexual partner within 5 years of age than five years older [OR = 0.53 (95% CI: 0.32–0.87), *p* = 0.012], having tertiary level education than primary education [OR = 0.11 (95% CI: 0.03–0.44), *p* = 0.002], low risk alcohol use [OR = 0.19 (95% CI: 0.04–0.78), *p* = 0.022] and having one sexual partner compared to two or more partners [OR = 0.43 (95% CI: 0.20–0.91), *p* = 0.028].

**Fig. 2:**
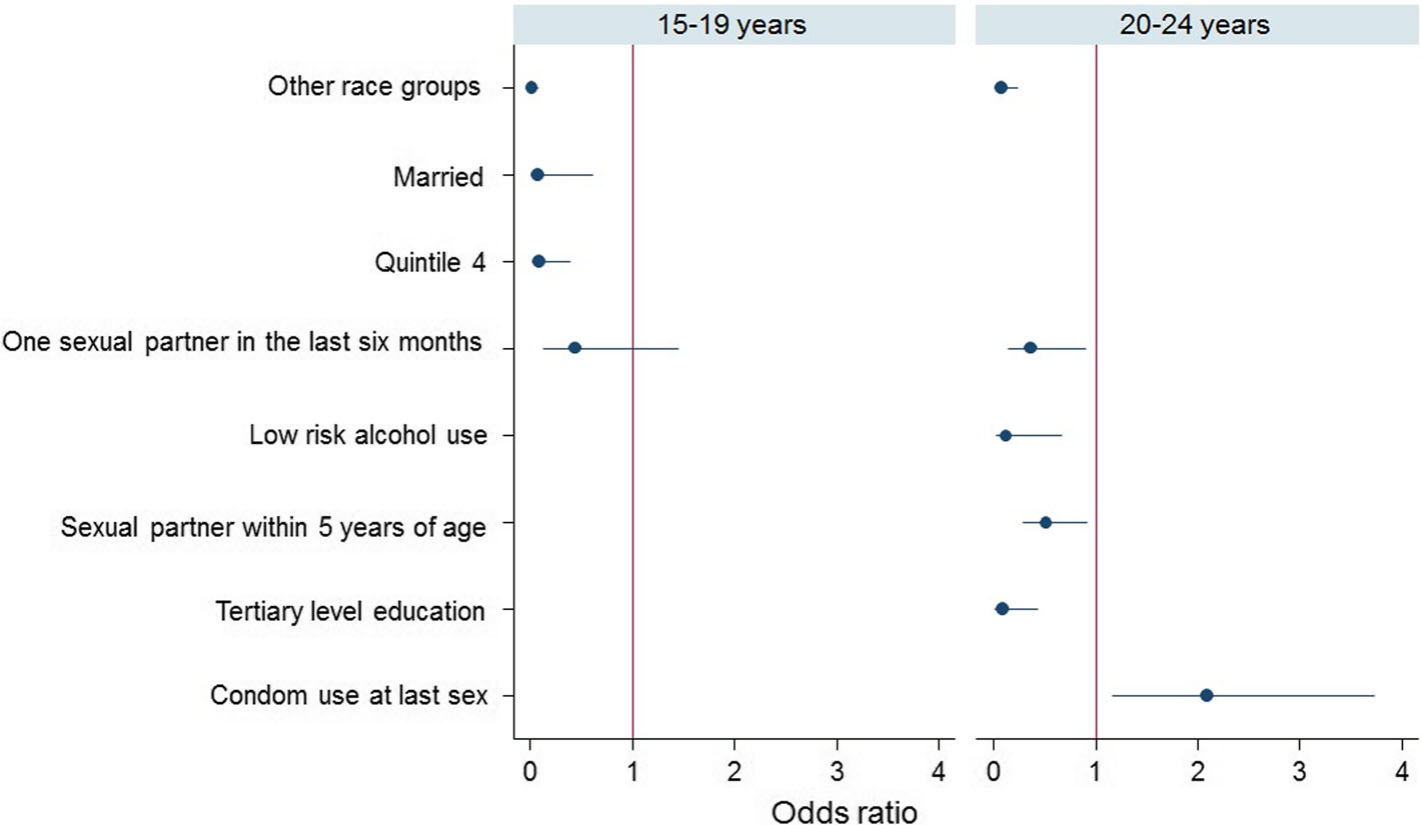
Multivariate logistic regression models (significant at *p* < 0.05) stratified by age for adolescent girls (15–19 years) and young women (20–24 years)

Figure 2 shows multivariate models for adolescent girls (15–19 years) and young women (20–24 year). In the adolescent girls model only race, marital status, and asset based SES were selected. Decreased likelihood of HIV infection was significantly associated with belonging to other race groups [OR = 0.01 (95% CI: 0.00–0.07), *p* < 0.001], being married [OR = 0.07 (95% CI: 0.01–0.60), *p* = 0.016], and living in less poor households (quintile 4 SES households) than most poor households (quintile 1 SES households) [OR = 0.08 (95% CI: 0.02–0.39), *p* = 0.002].

In the young women model race, alcohol use, age-disparate sexual partnership, educational level, and condom use at last sex were selected. Increased likelihood of HIV infection was only significantly associated with condom use at last sex [OR = 2.09 (95% CI: 1.17–3.73), *p* = 0.013]. Decreased likelihood of HIV infection was significantly associated with belonging to other race groups [OR = 0.17 (95% CI: 0.08–0.34), *p* < 0.001], having one sexual partner [OR = 0.6 (95% CI: (0.43–0.91), *p* = 0.014] low risk alcohol use [OR = 0.17 (95% CI: 0.08–0.34), *p* < 0.001], having a sexual partner within 5 years of age [OR = 0.29 (95% CI: (0.10–0.84), *p* = 0.022], and having tertiary level education, [OR = 0.29 (95% CI: (0.10–0.84), *p* = 0.022].

## Discussion

In South Africa, several strides have been made in the fight against HIV. Despite progress in the fight against HIV/AIDS, AGYW remain at great risk of new HIV infections. Therefore preventing HIV acquisition in this group remains a public health imperative and improved understanding of the drivers of HIV in young women is critical for developing appropriate interventions. Consequently, studies have sought to characterize social, behavioural and structural factors associated with HIV risk in young women [17, 18]. The current study found that young women 20–24 years old have a higher prevalence of HIV compared to adolescent girls 15–19 years old. This is in agreement with observation of other studies in South Africa and other Southern African counties [17, 18].

The regression analysis also confirmed that young women who are mainly Black Africans were at increased risk of HIV infection compared to adolescent girls. Generally in Southern Africa studies have shown that the road from adolescence (15–19 years) to young adulthood (20–24 years) is particularly perilous for young women as the risk that they will become infected with HIV is high [17]. This is mainly because as young women progress through this transitional period (i.e., time of rapid physical, psychological, and social development) the great majority become sexually active and engage in behaviours that put them at risk for HIV [18].

The findings showed that marriage has a protective effect against HIV infection especially among adolescent girls. Some studies have suggested that married adolescents are often at low risk for HIV infection, specifically, when both partners are uninfected at the time of marriage and subsequently engage in sexual activity exclusively with each other [19]. However, other studies have suggested that marriage may increase the vulnerability female adolescents to HIV infection due to limited bargaining power within the marriage and/or exposure to unprotected sex due to the desire for pregnancy [18]. Therefore, more studies are needed on factors that may increase or reduce risk for HIV among married female adolescents, especially because most are at child bearing age and more prone to pregnancy which is a critical phase for prevention of mother to child transmission.

In addition, living in least poor households was found to be protective against HIV infection for adolescent girls. Other studies also show that risk of HIV infection among young women and especially among adolescents is associated with socio-economic position [13, 14, 20]. Poor women are often economically dependent on men, and the need for economic support make it difficult for young women to insist on safer sexual practices [20]. This drives adoption of high-risk behaviours for HIV infection, which include early sexual debut, multiple sexual partnerships, limited condom use intimate partner violence, transactional and intergenerational sex [7–9, 20]. This underscores the need to address socio-economic drivers of the HIV epidemic among adolescent girls though structural programmes that targets poor and vulnerable households to reduce economic vulnerability and HIV.

Furthermore, reported condom use at last sex was associated with increased risk of HIV among young women. There is evidence that condom use reported by young people engaging in risky sexual behaviour is not high enough to curb the spread of HIV [2]. Evidence also suggests that those who reported not using condoms at last were inconsistent condom users and that if the reported condom use at last sex is not consistent reduction in risk of HIV infection will also not be consistent [21]. Therefore, prevention of HIV risk among women should be promoted through correct and consistent condom use.

The study found that tertiary education has a protective effect against HIV among young women. It has been hypothesized that education prepares individuals better to mount a response to the HIV/AIDS epidemic. Particularly, because higher levels of education may provide a framework of knowledge and understanding of causality into which HIV prevention messages can be assimilated [22, 23]. Evidence shows that educated women or individuals with higher level of education are better equipped, or empowered to change their sexual behaviour, adopt safe sexual practices [11, 22–24]. There is therefore a need to promote access and equity to education as an important foundation for effective HIV prevention response among AGYW.

The findings also showed that having a sexual partner within the same age range was protective of HIV infection among young women. Several other nationwide surveys in South Africa have shown that young people whose sexual partners are several years older than themselves are usually at higher risk of acquiring HIV than those whose partners are age matched [3, 25–27]. Similar observations have also been made in Swaziland, Tanzania and Zimbabwe where studies showed that the larger the age-gap between sexual partners, the greater the likelihood of being HIV-infected [28]. Such age differentials reinforce gendered power dynamics impacting on the ability to decide whom they have sex with and the type of sex, and whether that sex is protected, which increase young women’s vulnerability to HIV [18, 29, 30].

Low risk alcohol drinking was also found to have a protective effect against HIV infection among young women. Studies have shown than risky drinking is associated with unsafe sex such as the ability to use condoms [10]. Furthermore, evidence shows that young people who consume alcohol are more likely experience their first sexual encounter at a younger age, and engage in sex with multiple partners [31, 32]. Reducing risky alcohol consumption should be encouraged as part of the HIV prevention strategies for reducing risk of among young women.

### Limitations of the present study

Behaviour related variables are based on self-report and these may be affected by both recall and social desirability bias. The exclusion of other unmeasured potential covariates in the analysis could be another limitation. Furthermore, the cross-sectional nature of the study means that it is only limited to assessing associations and cannot therefore infer any causality. Nevertheless, the study is nationally representative and the finding can be generalized to AGYW age 15–24 years in the country.

## Conclusions

The findings of this study highlight the need to address the structural socio-economic drivers of the HIV epidemic among AGYW by targeting those that come from the poor and most vulnerable households. In addition, there is a need to promote education as well as equity and access to schooling, and intensify efforts targeting risk behaviours such age disparate sexual relationships, inconsistent condom use and risky alcohol consumption. Pivotal to the success of such interventions is the need to address societal norms supportive of male superiority and sexual entitlement, leading to gender inequality causing females to be unable to negotiate safe sex.

## Abbreviations

AGYW: Adolescent girls and young women
ARV: Antiretroviral
AUDIT: Alcohol Use Disorder Identification Test
CI: Confidence intervals
DBS: Dried blood spots
EAs: EAs-enumeration areas
EIA: Enzyme immunoassay
HCT: HIV testing and counselling
HIV: Human immunodeficiency virus
MCA: Multiple correspondence analyses
OR: Odds ratios
SES: Socio-economic status
USA: United States of America

## References

[1] UNAIDS (2016). HIV prevention among adolescent girls and young women: putting HIV intervention among adolescent girls and young women on the fast-track and engaging men and boys.

[2] UNAIDS (2015). Global AIDS update 2016: fast-tracking the response in eastern and southern Africa – focus on Adolescent Girls & Young Women. Global AIDS update 2016, and prevention gap report, UNAIDS, Geneva; 2016.

[3] Shisana O, Rehle T, Simbayi LC, Zuma K, Jooste S, Zungu N (2014). South African national HIV prevalence, incidence and behavior survey, 2012.

[4] Niëns L, Lowery D (2009). Gendered epidemiology: sexual equality and the prevalence of HIV/AIDS in sub-Saharan Africa. Soc Sci Q.

[5] Wilson CM, Wright PF, Safrit JT, Rudy B (2010). Epidemiology of HIV infection and risk in adolescents and youth. J Acquir Immune Defic Syndr.

[6] Pettifor AE, Rees HV, Kleinschmidt I, Steffenson AE, MacPhail C, Hlongwa-Madikizela L (2005). Young people's sexual health in South Africa: HIV prevalence and sexual behaviors from a nationally representative household survey. AIDS.

[7] Pettifor A, MacPhail C, Rees H, Cohen M (2008). HIV and sexual behavior among young people: the south African paradox. Sex Transm Dis.

[8] Jewkes RK, Dunkle K, Nduna M, Shai N (2010). Intimate partner violence, relationship power inequity, and incidence of HIV infection in young women in South Africa: a cohort study. Lancet.

[9] Stöckl H, Kalra N, Jacobi J, Watts C (2013). Is early sexual debut a risk factor for HIV infection among women in sub-Saharan Africa? A systematic review. Am J Reprod Immunol.

[10] Kalichman SC, Simbayi LC, Kaufma M, Cain D, Jooste S (2007). Alcohol use and sexual risks for HIV/AIDS in sub-Saharan Africa: systematic review of empirical findings. Prev Sci.

[11] Glynn JR, Carael M, Buve A, Anagonou S, Zekeng L, Kahindo M (2004). Does increased general schooling protect against HIV infection? A study in four African cities. Tropical Med Int Health.

[12] Doyle AM, Mavedzenge SN, Plummer ML, Ross DA (2012). The sexual behaviour of adolescents in sub-Saharan Africa: patterns and trends from national surveys. Tropical Med Int Health.

[13] Mbirimtengerenji ND (2007). Is HIV/AIDS Epidemic Outcome Of poverty in sub-Saharan Africa?. Croat Med J.

[14] Mufune P (2014). Poverty and HIV/AIDS in Africa: specifying the connections. Social Theory & Health.

[15] Booyson F, van der Berg S, Burger R, von Maltitz M, du Rand G (2008). Using an asset index to assess trends in poverty in sevens sub-Saharan African countries. World Dev.

[16] Saunders JB, Aasland OG, Babor TF, De la Fuente JR, Grant M (1993). Development of the alcohol use disorders identification test (AUDIT): WHO collaborative project on early detection of persons with harmful alcohol consumption-II. Addiction.

[17] Harrison A, Colvin CJ, Kuo C, Swartz A, Lurie M (2015). Sustained high HIV incidence in young women in southern Africa: social, behavioral and structural factors and emerging intervention approaches. Current HIV/AIDS Reports.

[18] Dellar RC, Dlamini S, Karim QA (2015). Adolescent girls and young women: key populations for HIV epidemic control. J Int AIDS Soc.

[19] Clark S. Bruce J, Dude A. Protecting Young Women from HIV/AIDS: the case against child and adolescent marriage. Int Fam Plan Perspect 2006; 32(2):79–88. 10.1363/3207906 16837388

[20] Rodrigoa C, Rajapakseb S (2010). HIV, poverty and women. International Health.

[21] Katz I, Low-Beer D (2008). Why has HIV stabilized in South Africa, yet not declined further? Age and sexual behavior patterns among youth. Sex Transm Dis.

[22] Baker DP, Leon J, Collins JM (2011). Facts, attitudes, and health reasoning about HIV and AIDS: explaining the education effect on condom use among adults in sub-Saharan Africa. AIDS Behav.

[23] Jukes M, Simmons S, Bundy D (2008). Education and vulnerability: the role of schools in protecting young women and girls from HIV in southern Africa. AIDS.

[24] Michelo C, Sandøy IF, Fylkesnes K (2006). Marked HIV prevalence declines in higher educated young people: evidence from population-based surveys (1995–2003) in Zambia. AIDS.

[25] Shisana O, Simbayi LC. Nelson Mandela/HSRC study of HIV/AIDS: South Africannational HIV prevalence, behavioural risks and mass media: household survey 2002: HSRC Press; 2002.

[26] Shisana O. South African national HIV prevalence, HIV incidence, behaviour and communication survey, 2005: HSRC press; 2005.

[27] Shisana O, Rehle T, Simbayi L, Zuma K, Jooste S. South African national HIV prevalence incidence behaviour and communication survey 2008: a turning tide among teenagers? 2009.

[28] UNICEF. Opportunity in crisis: preventing HIV from early adolescence to young adulthood: UNICEF; 2011.

[29] Leclerc-Madlala S (2008). Age-disparate and intergenerational sex in southern Africa: the dynamics of hypervulnerability. AIDS.

[30] Leclerc-Madlala S, Simbayi LC, Cloete A. The sociocultural aspects of HIV/AIDS in South Africa. HIV/AIDS in South Africa 25 Years On: Springer; 2009. p. 13–25.

[31] Pithey A, Parry C (2009). Descriptive systematic review of sub-Saharan African studies on the association between alcohol use and HIV infection. Journal of Social Aspects of HIV/AIDS.

[32] Hahn JA, Woolf-King SE, Muyindike W (2011). Adding fuel to the fire: alcohol’s effect on the HIV epidemic in sub-Saharan Africa. Current HIV/AIDS Reports..

